# On the Treatment of Laryngeal Growths

**Published:** 1889-06

**Authors:** Barclay J. Baron

**Affiliations:** Physician in charge of the Throat Department of the Bristol General Hospital


### ON THE TREATMENT OF LARYNGEAL GROWTHS.

BY

BARCLAY J. BARON, M.B. Edin.;

*Physician in charge of the Throat Department of the Bristol General Hospital.*

---

HAVING, during the past six years, had many opportunities of seeing cases in which symptoms of laryngeal trouble were present, and in which, on laryngoscopic examination, outgrowths in that part of the respiratory tract were found, I think that, without pretending to say anything new, I may still say a few words that are not altogether devoid of value.

Deferring for the moment the consideration of malignant growths of the larynx, we have to discuss all other forms of outgrowth from the mucous membrane found in that organ.

In order still further to simplify matters, I will separate all those excrescences which are met with in syphilis, phthisis and lupus from those which have no such origin, and call the latter benign or innocent growths.

As regards the ætiology of benign laryngeal tumour formations, there is no doubt whatever but that people, who either from the nature of their business, or voluntarily submit their vocal organs to considerable strain, are infinitely more liable to be attacked by them than others who use their voices in moderation, and rarely, or never, for other purposes than conversation. In fact, just as no hypertrophy of the muscles of a limb takes place under steady, quiet, long-continued work—*e.g.* writing, every day during many years, causes no increase in the bulk of the muscles of the arm, there being no hyperæmia of the limb set up by, and necessary for, the doing the work which is required, and yet the limb quickly gets larger under the intermittent strain of hard muscular work—so no amount of conversation causes laryngeal hyperæmia, and yet a moderate amount of singing will quickly do so in the same person, in many cases.

Therefore, the same class of patients who consult us for catarrh of the larynx, will present to us cases of the disease under consideration, *e.g.* actors, singers, school teachers—especially those who teach some rudimentary singing—and drilling masters, in my own practice.

We shall find, too, that using the voice in an atmosphere as of a smoking concert, charged with irritating fumes, or with dust and burnt gas as in a theatre, &c., predispose to these outgrowths.

As regards the ages of my cases, I have seen none under twenty, and have seen one case over sixty years old. The males were largely in excess of females.

The most noticeable symptom, and the one which induces patients to consult a medical man, is usually alteration in the voice, which becomes hoarse, and may be in some cases nearly extinguished; and patients who continue to use their voices as long as they are only *slightly* hoarse, during which time there has doubtless been congestion of the larynx, are obliged to seek advice, because the hoarseness has increased, and then, on laryngoscopic examination, a growth or growths can be seen. Rarely do we notice pain, or interference with deglutition or respiration; but patients often complain of a tickling hard cough, with the expectoration of small pellets of mucus, similar to that which is characteristic of irritation of the larynx or trachea.

The following case will demonstrate most clearly the dependence of these growths on chronic congestion:

T. W., aged 30, who had for fifteen years got his living by singing in public-houses, came under my care, at the Bristol General Hospital, suffering from chronic laryngitis. In the summer of 1887 he was dismissed, cured, after repeated painting of the vocal cords, which were red and thickened, with solutions of chloride of zinc in glycerine.

I lost sight of him for about ten months, when he returned, complaining of hoarseness, inability to sing long without becoming much fatigued, his throat aching, and his voice almost failing; these being the symptoms of which he had complained in the previous year, I expected to find a renewal of the chronic catarrh of the larynx, instead of which I discovered a small smooth red growth, attached by a pedicle to the left vocal cord near its anterior extremity, with some congestion of the vocal cord of the same side, and with a similar condition of the right cord, against which the polypus impinged on phonation.

Here, then, certainly not more than ten months were sufficient to give rise to a distinct growth, as the result of chronic congestion of the parts, due to straining the voice, in a man who sang whilst he was hoarse, and therefore unfit, and in an atmosphere charged with the irritating fumes of tobacco and alcohol.

I advised removal, which could have been done either with the snare, forceps, or Voltolini's sponge; but the patient would not submit, and I have not seen him since.

This is an instance of a single pedunculated growth, in which operation would have at once restored normal voice: but we frequently see cases in which the growths are sessile and multiple; then the treatment must be different.

The two following cases, which are selected from amongst a number, will illustrate the pathological condition and its remedy:

A. C., aged 21, a Board School mistress, came under my care in January, 1887, with complete aphonia of some months standing. On laryngoscopic examination I found the anterior half of her left vocal cord covered by an irregular, sessile, pink-coloured growth, which projected into the glottic chink, and on attempted phonation touched the opposite cord and caused the aphonia, of which she complained.

I painted this growth with solutions of chloride of zinc in glycerine grs. x. to xxx. to the ounce, perchloride of iron solutions, and, lastly, lactic acid, beginning with 50 per cent. solution and leaving off with 80 per cent. solution. The result was that she regained her voice, was only very slightly hoarse, and ceased attending the hospital, as she got a situation as head mistress of a school, and daily submitted her voice to considerable strain. The growth was still to be seen, but had receded from the free edge of the vocal cord, having, in fact, shrunk under the action of the astringents used.

In June, 1888, she came to see me again because of slight hoarseness, and I found the left vocal cord just as it was on her last visit to the Hospital, nine months previously; the right vocal cord was thickened, and after painting this with the same astringents as had previously been so efficacious, she quickly got well, and I have not seen her since.

M. C., a retired sergeant-major, consulted me for hoarseness, which was so pronounced as to prevent him giving the word of command in drilling boys and girls in schools: in fact, it was with some difficulty that I could understand some of his words, his deep bass voice sounded so oddly.

On examination, I found his right vocal cord covered in its whole length, with a fleshy-looking irregular growth, which projected well into the glottic opening, the other cord being congested.

Inhalations of compound tincture of benzoin, cold compresses at night, and systematic painting of the growth with solutions of chloride of zinc and perchloride of iron were productive of such a good result, that his voice became clearer than it had been for years, the outgrowth meanwhile having steadily diminished in size, and having changed in appearance, as it looked pale and shrunken.

In such cases as these, with a diffused growth or rather series of growths, which are sessile, removal by instruments is often quite impossible, but frequent application of astringents such as I have named, compresses, inhalation of vapor benzoin, and rest of the voice will prove of the greatest value.

Another class of case, of which the following is an example, shows the advantage of a somewhat different method of treatment:

M. W., aged 28, a schoolmaster, consulted me in September, 1888, for hoarseness, which was so considerable as to threaten to deprive him of his means of earning a livelihood. On examining his larynx, I found a small purple-coloured growth, about the size of a large hemp-seed, and as irregular on its surface as a mulberry, attached by a broad base to the anterior third of the left vocal cord, near the angle of insertion of the cords into the thyroid cartilage. I thoroughly rubbed this growth with a hard substance and applied chloride of zinc solution, which removed the growth, and he regained his voice.

He came up to see me a few days ago, eight months after the operation, and I could just distinguish a small stump, of grey colour, in the position that the growth formerly occupied, and although in the interval he had submitted his voice to great strain, it had borne it exceedingly well, there being merely a faint trace of hoarseness after prolonged effort.

Here, then, the growth, which would have been exceedingly difficult to reach and grasp with either snare or forceps, was rubbed off, and the astringent solution applied to the raw surface. So far, there has been no sign of recurrence. The treatment of these pathological new formations, therefore, must be varied as the growth is pedunculated or sessile, and single or multiple; but just as, only by accurate laryngoscopic examination can we ascertain their presence, so it is only by painstaking and accurate local treatment, always with the aid of the laryngeal mirror, that we can hope to eradicate them and cure our patients.

As regards the outgrowths which we sometimes see in cases of phthisis laryngea, which are commonly found on the inter-arytenoid mucous membrane, and usually only in that situation, we generally see other signs of the grave constitutional mischief underlying them, in the shape of infiltration of the mucous membrane covering and in the neighbourhood of the arytenoids, of the ventricular bands and side-walls of the larynx, of the epiglottis, or of the vocal cords, with or without ulceration. The lungs usually afford corroborative diagnostic evidence, primary phthisis laryngea being of the greatest rarity, if even it ever occurs. Removal of such growths is, in the great majority of cases, contra-indicated; as, in my opinion, the less a phthisical larynx is worried by local treatment, especially in the pre-ulcerative stage, the better. After ulceration has ensued, the question of the propriety of scraping the parts infiltrated with tubercle, and rubbing into the raw surface strong solutions of lactic acid, must be debated: I have certainly seen this treatment, and this *alone*, improve the appearance of the diseased parts, and even bring about cure, with cicatrisation of the tubercular ulcer, especially in the pharynx, in rare instances.

As to iodoform, iodol, carbolic-acid spray, menthol, *et hoc genus omne*, I do not see any cause to look on them as curative agents, and some of them are not even palliatives in laryngeal phthisis.

Syphilis sometimes, and especially in its tertiary stage, leads to considerable overgrowth of mucous membrane, which is in round projections or lumps, which cause great deformity of the larynx, and may so block the glottis as to render tracheotomy a vital necessity.

Such growths cannot, as a rule, be interfered with, with much hope of doing good, as the resulting cicatrisation will quite, or more than, compensate for the space opened up by operation.

After tracheotomy, however, attempts may be made, by cutting and dilatation, to open up the blocked passage, and allow the patient to breathe *per vias naturales*; but, it must be admitted, often without success. As a rule, most of such cases are too far advanced for iodides to be of value, but I have seen, even in an apparently hopeless and long-standing case, some lessening of the growth take place when they are given in large doses; of course, in secondary conditions, much may be done by this drug and mercury.

There is also no doubt but that tracheotomy, by giving rest to the narrowed and over-worked, irritated glottic structures, is of great value in these cases. I have a patient at present under my care, in whom there has been a distinct widening of the chink since the operation was performed. Although the tube has to be worn permanently by many of these patients, yet the benefit to the general health obtained by ensuring the free entry of air into the lungs, quiet refreshing sleep, and freedom from the anxiety caused by the constant dread of suffocation, under which they labour, makes the operation justifiable, long before a considerable encroachment on the glottic chink is present.

With reference to malignant laryngeal growths, I am thankful to say that I have not had to assume entire responsibility of treating many cases, and I only know the results of radical measures from reading the accounts of published cases.

From what I have seen, I do not consider that tracheotomy is done early enough in these patients, because, whilst cure cannot be dreamt of from the operation, the rest to the diseased structures and the retention of good general health, obtained by opening up a channel for an abundant air-supply, will, there is reason to believe, defer the stage of ulceration, of secondary infiltration, and of a fatal termination.

The feeding of these patients, especially during the intercurrent attacks of perichondritis which come on, is of the greatest importance. Liquids, as a rule, are not easily swallowed, as the epiglottis being thickened and erect, they tend to "go the wrong way." I always order them to be thickened by arrowroot, beaten-up egg, &c.; and I find that curds and whey, custard, blanc mange, beef and chicken jelly, raw eggs bolted almost whole, bread and milk made like baby's pap, egg and milk with or without alcohol, and all of these in bad cases, taken after a 5 to 10 per cent. solution of cocaine has been carefully painted on the diseased structures, are well borne.

The food should not be sipped, but bolted in good quantity, as there is not so much pain in swallowing, at one gulp, two or three ounces of thickened milk as a tablespoonful. Naturally, rectal alimentation also has to be thought of; and the value of cocaine, accurately applied with a brush, under the guidance of the mirror, and not vaguely sprayed into the throat, is extremely great in quelling pain and promoting comfort, sleep, &c.

